# Effect of Vitamin D supplementation on reduction in levels of HbA1 in patients recently diagnosed with type 2 Diabetes Mellitus having asymptomatic Vitamin D deficiency

**DOI:** 10.12669/pjms.334.12288

**Published:** 2017

**Authors:** Fawad Ahmad Randhawa, Saqib Mustafa, Dur Muhammad Khan, Shahid Hamid

**Affiliations:** 1 Dr. Fawad Ahmad Randhawa, FCPS, MCPS (Med.), FCPS (Endocrinol.). Department of Medicine, King Edward Medical University Lahore Pakistan; 2 Dr. Saqib Mustafa, Department of Medicine, King Edward Medical University Lahore Pakistan; 3 Dr. Dur Muhammad Khan, FRCP. Department of Medicine, King Edward Medical University Lahore Pakistan; 4 Dr. Shahid Hamid, FCPS. Department of Medicine, King Edward Medical University Lahore Pakistan

**Keywords:** Blood sugar level, Metformin, Type II diabetes mellitus, HbA1c, Vitamin D supplementation

## Abstract

**Objective:**

To study the effect of Vitamin D supplementation on reduction in level of HbA1 in patients recently diagnosed with diabetes mellitus Type II having asymptomatic Vitamin D deficiency.

**Methods:**

This randomized control trial was conducted at East Medical Ward Mayo Hospital Lahore for 6 months from January 01 2016 to June 30, 2016. 114 Patients were included through non probability purposive sampling technique. Informed consent and demographic information was collected. Patients were divided in two groups by randomization through tossing a coin. Group-A patients received Metformin tablet alone at 500 mg after dinner and Group-B patients were treated with same dosage of Metformin along with oral vitamin D at 200,000 IU monthly for three months. Blood sample was obtained at baseline, 3 months and 6 months of initiation of therapy. All samples were sent to the laboratory for complete blood count, blood sugar fasting, serum calcium, serum phosphorous, serum alkaline phosphatase, HbA1c and serum 25 Hyroxy Vitamin D (S-25(OH) D) levels and iPTH. Data entry and analysis was done by using SPSS 20.

**Results:**

The mean age of patients in metformin group was 42.37±4.59 years while mean age of patients in combination group was 43.33±4.86years. Males were 45.6% and females were 54.4% in metformin group while in combination group, 36.8% were males’ and 63.2% were females’. At baseline, in metformin group, mean Vitamin D level was 17.09±1.73mg/dl and in combination group, mean Vitamin D level was 16.49±1.56mg/dl. The difference was insignificant (p>0.05). On 2nd visit, combination group mean Vitamin D was 29.04±3.96mg/dl. At baseline, 1^st^ and 2^nd^ visit, in metformin group, mean HbA1c was 7.59±0.47%, 7.46±0.25% and 7.30±0.29%. At baseline, 1^st^ and 2^nd^ visit, in combination group, mean HbA1c was 7.71±0.19%, 7.57±0.21% and 7.43±0.26%. The difference was insignificant (p>0.05) at baseline while significant on later follow-ups (P<0.05).

**Conclusion:**

Vitamin D supplementation improved the glycemic control but substantial reduction in HbA1c was statistically insignificant in both groups.

## INTRODUCTION

Vitamin D deficiency is commonly due to reduced exposure to ultraviolet-B (UVB) radiation, and it is observed that there is reduced production of Vitamin D in the skin cells.^1^ Vitamin D has considerable importance in public health, because Vitamin D deficiency is widespread and is frequently related with musculoskeletal diseases.^2^

UVB emission is connected with a 162% increase in plasma 25 hydroxyvitamin D (25[OH] D) absorptions.^3^ There is an assumption that daily an intake of Vitamin D 1,000 IU (25 ug) can increase 25(OH) D levels of approximately 10 ng/ml (25 nmol/l).^4^ Oral intake Vitamin D 1,500 IU every day, 10,500 IU once weekly, or 45,000 IU once every 28 days has been manifested to outcome in parallel growth of 15–16 ng/ml (37.4–40.0 nmol/l) in 25(OH) D levels.^5^

During last two decade, There are many Vitamin D alpha receptors have been developed in various tissues of our body and many new activities of Vitamin D have been studied ruminated by these receptors.^6^ Vitamin D status can also comprise a significant function in glucose homeostasis, progression of metabolic syndrome as well as Type 2 diabetes.^7^ Researches have proved that Vitamin D affects insulin secretion along with tyrosine phosphorylation of the insulin receptor.^8^ It is observed that short serum 25-hydroxyVitamin D (25(OH)D) levels are connected with major adverse cardiovascular occurrence, increase insulin confrontation, cancers, and increase mortality rate, at least in subjects through metabolic syndrome.^9^

That is why adequate 25(OH)D levels are connected with a lesser possibility of confrontation of diabetes.^10^ It has also been reported that most of the patients suffering from Type 2 diabetes have low 25(OH)D levels and they have contrary connection with hemoglobin A1c (HbA1c) levels and Vitamin D status.^11^ Keeping in mind these findings, one can hypothesize that Vitamin D supplements decrease insulin resistance and reduces HbA1c levels in victims with diabetes.^12^

However, supplementation studies have not unambiguously found that Vitamin D favors an improvement in glucose homeostasis parameters.^13^ That is why this study was planned and designed for our population of newly diagnosed type 2 diabetics to regulate the part of Vitamin D supplements in HbA1c reduction. This also provides useful data regarding efficacy of Vitamin D supplements in management of diabetes Type II as well as opens new gates and forums of discussion regarding management of type 2 diabetics.^14^

## METHODS

After approval of synopsis from board of study and Institutional Review Board and Advanced Study Review Board of King Edward Medical University, all 114 patients were randomized to either Group A (Patients were treated with Metformin only at 500 mg after dinner) or B (Patient were treated with Metformin same dosage and oral Vitamin D supplementation 200,000 IU/month for 3 months) after informed consent from patients or attendants. Patients with age 35-50 years of either gender who were diagnosed cases of type 2 diabetes mellitus having clinically asymptomatic serum vitamin D levels below 20ng/ml and taking metformin only for the treatment of type 2 diabetes mellitus with HbA1C level in between 7-9 were included in the study.

All the patients who had fasting serum calcium >10.5 mg/dL, history of diseases like nephrolithiasis, hypercalciuria, malignancy, tuberculosis, sarcoidosis, Paget’s disease, malabsorption syndromes on medical records, patients of renal insufficiency diagnosed on the basis of renal profile, presence of proximal myopathy and pregnant patients were excluded.

Demographic information (like name, age, sex, height and weight) was also obtained. Blood sample was obtained from each patient at baseline and was sent to the laboratory for complete blood count, blood sugar fasting, serum calcium, serum phosphorous, serum alkaline phosphatase, HbA1c and S-25(OH) D levels and iPTH. At the end of 3^rd^ month (at first follow up), HbA1C levels were measured in all patients and S-25(OH) D levels were measured only in Group-B patients that received oral vitamin D. S-25(OH) D levels of over 150 ng/ml was not found in any patient at follow-up so no patient was dropped out of the study. At the end of 6^th^ month(at final follow up), blood sample was obtained from each patient and was sent to the laboratory for HbA1c and S-25(OH)D levels were measured only in Group-B patients.

All this information was recorded on predesigned proforma. Data entry and analysis was done by using SPSS 20. Quantitative variables (Age, Height, Weight, Blood Sugar Fasting, Serum Calcium, Serum Phosphate, Serum Alkaline Phosphate, HbA1C, S-25(OH) D, CBC, RFT, and LFT) were presented by using mean± SD. Qualitative variables (Gender, Efficacy) were presented by using frequency table and percentages. Repeated measure ANOVA/Fried Man test was used to see the HbA1C level in both groups during course of follow up time period. Paired sample t-test/Wilcoxson signed ranked test was used to compared the HbA1C level before and after treatment. Efficacy of treatment was assessed in both treatment groups by using Chi-Square test. P-value < 0.05 was taken significant.

## RESULTS

The mean age of patients in metformin group was 42.37±4.59 years while mean age of patients in combination group was 43.33±4.86years. There were 26 males (45.6%) and 31 females (54.4%) in metformin group while in combination group, there were 21 males (36.8%) and 36 females (63.2). At the baseline, in metformin group, there were 24 patients on anti-glycemic treatment while in combination group; there were 33 patients on anti-glycemic treatment. In metformin group, mean height was 1.68±0.08m and in combination group, mean height was 1.74±0.05m. In metformin group, mean weight was 72.08±11.10 kg and in combination group, mean weight was 70.61±9.65 kg. In metformin group, mean BMI was 25.49±3.99kg/m^2^ and in combination group, mean BMI was 23.50±3.68 kg/m^2^. In metformin group, mean duration of diagnosis of diabetes was 2.63±1.26 years and in combination group, mean duration of diagnosis of diabetes was 3.25±1.18 years. At baseline, in metformin group, mean BSR was 219.88±40.40mg/dl and in combination group, mean BSR was 240.40±37.11mg/dl. The difference was significant (p 0.0065). At 1^st^ visit, in metformin group, mean BSR was 184.37±34.08mg/dl and in combination group, mean BSR was 195.21±30.98mg/dl. The difference was insignificant (P 0.078).

In 2^nd^ visit, in metformin group, mean BSR was 165.12±21.65mg/dl and in combination group, mean BSR was 168.42±19.89mg/dl. The difference was insignificant (P 0.399). Within groups, there was significant decrease in BSR (P 0.00). At baseline, in metformin group, mean calcium was 9.21±0.79mg/dl and in combination group, mean calcium was 9.15±0.74mg/dl. The difference was insignificant (P 0.668). At 1^st^ visit, in metformin group, mean calcium was 9.45±0.80mg/dl and in combination group, mean calcium was 9.23±0.74mg/dl. The difference was insignificant (P 0.128). In 2^nd^ visit, in metformin group, mean calcium was 9.79±0.81mg/dl and in combination group, mean calcium was 9.90±0.66mg/dl. The difference was insignificant (p 0.448). Within groups, there was significant increase in calcium (P 0.00). At baseline, in metformin group, mean phosphorus was 3.44±0.85mg/dl and in combination group, mean phosphorus was 3.37±0.91mg/dl. The difference was insignificant (P 0.663). At 1^st^ visit, in metformin group, mean phosphorus was 3.79±0.91mg/dl and in combination group, mean phosphorus was 3.88±1.03mg/dl. The difference was insignificant (P 0.632).

In 2^nd^ visit, in metformin group, mean phosphorus was 4.16±1.09mg/dl and in combination group, mean phosphorus was 4.34±1.16mg/dl. The difference was insignificant (P 0.390). Within groups, there was significant increase in phosphorus (P 0.000). At baseline, in metformin group, mean AP was 98.26±25.09mg/dl and in combination group, mean AP was 98.47±30.59mg/dl. The difference was insignificant (P 0.968). At 1^st^ visit, in metformin group, mean AP was 97.26±29.02mg/dl and in combination group, mean AP was 95.19±30.99mg/dl. The difference was insignificant (P 0.713). In 2^nd^ visit, in metformin group, mean AP was 93.93±31.41mg/dl and in combination group, mean AP was 99.14±29.59mg/dl. The difference was insignificant (P 0.364). Within metformin group, there was insignificant decrease in AP (P 0.442), and in combination group, AP there was insignificant increase in AP (P 0.914).

**Fig. 1:**
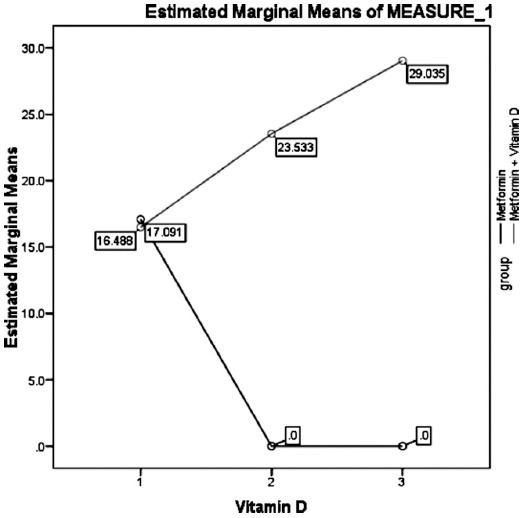
Comparison of Vitamin D in both groups at different follow-ups. Repeated measurements ANOVA was applied p-value = 0.000 (Significant)

**Fig. 2:**
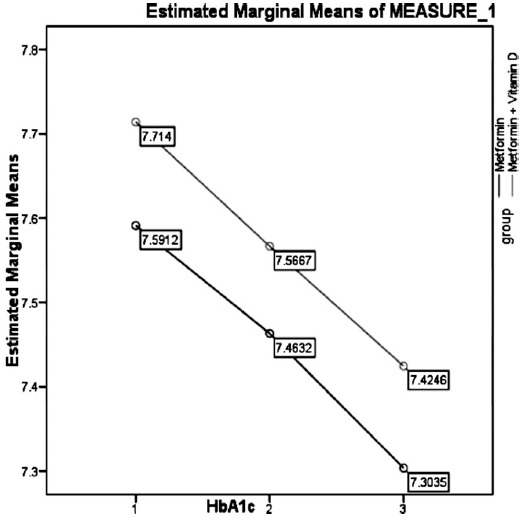
Comparison of HbA1c in both groups at different follow-ups. Repeated measurements ANOVA was applied p-value = 0.000 (Significant)

Tables I and II and Fig. 1 and 2 show comparisons of vitamin D levels and HbA1c in both groups at different follow ups respectively and ANOVA analysis.

**Table-I:** Comparison of Vitamin D in both groups at different follow-ups.

*Vitamin D level at*	*Group*		*p-value*

	*Metformin*	*Metformin + Vitamin D*	
Baseline	17.09±1.73	16.49±1.56	0.053
1^st^ visit	0	23.53±3.16	NA
2^nd^ visit	0	29.04±3.96	NA
p-value	NA	0.000	

**Table-II:** Comparison of HbA1c in both groups at different follow-ups.

HbA1c at	*Group*		*p-value*

	*Metformin*	*Metformin + Vitamin D*	
Baseline	7.59±0.47	7.71±0.19	0.071
1^st^ visit	7.46±0.25	7.57±0.21	0.020
2^nd^ visit	7.30±0.29	7.43±0.26	0.021
p-value	0.000	0.000	

## DISCUSSION

Despite the suggested role of vitamin D in the prevention of diabetes and cardiovascular disease or its risk factors, the evidence is not consistent and there is a paucity of randomized controlled trials in this field. The results of this study were very conflicting when compared with other studies. One reason for conflicting results in this study could be explained by the fact that in HbA1c was significantly higher in combination group as compared to metformin alone group, that is why at final follow-up the mean HbA1c was also low in metformin group alone.

In Type II diabetes mellitus, significant improvements in HbA1c are obtained with enhanced Vitamin D supplementation as part of drug regimen over time. Investigation provides the first known evidence of a relationship between enhanced Vitamin D supplementation as part of a pre-existing medical regimen taken over long term and determinants of Type II diabetes mellitus in a group of overweight and obese patients with Type II diabetes mellitus.^15^

Experimental data have indicated that Vitamin D insufficiency has an important influence on glucose metabolism. The majority of epidemiological studies have demonstrated an association between low Vitamin D and insulin resistance and/or Type II diabetes mellitus. Nevertheless evidence from randomized controlled trials remains inconclusive.^16^

Nwosu et al showed that after three months of Vitamin D supplementation, there was a significant increase in 25(OH)D in Type II diabetes mellitus patients (p = 0.015). This study concluded that there was a clinically significant decrease in HbA1c from 8.5±2.9% at baseline to 7.7±2.5% in Type II diabetic patients. This study included both Type I and Type II diabetics and all the patients were given vitamin D and interestingly it also concluded that there was no clinically-significant decrease in HbA1c in Type I diabetics (8.5 ± 1.2 to 8.53 ± 1.1%).^17^

Another study aimed to investigate the effect of 16-week daily vitamin D3 supplementation on HbA1C. Interestingly this double-blind, randomized, placebo-controlled trial showed that despite the fact that 25(OH) D increased from a mean of approximately 29nmol/L at baseline to 49 nmol/L after intervention but did not improve HbA1c.^18^

The major contrast of this study with current one is that they included healthy subjects only and they replaced oral vitamin D daily in two dosage contraries to the present study, where vitamin D was given monthly.

The results of the current study are comparable with the Krul-Poel et al. who showed that mean baseline serum 25-hydroxyVitamin D increased from 60.6 ± 23.3 to 101.4 ± 27.6 nmol/L and 59.1 ± 23.2 to 59.8 ± 23.2 nmol/L in Vitamin D plus metformin group and metformin alone group, respectively. Mean baseline HbA1c was 6.8 ± 0.5% in both groups. After 6 months, no effect was seen on HbA1c (mean difference: b = 0.4 [95% CI20.6 to 1.5]; P = 0.42) and other indicators of glycemic control (HOMA of insulin resistance, fasting insulin, and glucose) in the entire study population. Subgroup analysis in patients with a serum 25(OH) D<50 nmol/L or anHbA1c level> 7% (53 mmol/mol) did not differ the results.^19^

Prakash et al., results state otherwise that judicious supplementation of vitamin D amongst vitamin D deficient type 2 diabetic patients with near normal HbA1c can reduce the blood sugar level. HbA1c reduce to target levels if it is minimally elevated. These conflicting results can be explained by the fact that in this study conducted by Prakash et al. they did not alter the medicines in any group and most of the study subjects were taking more than one antidiabetic medicines. Moreover, the replacement oral vitamin D supplementation done was at dose of 60.000IU weekly for 3 months in one group as compared to present study where it was given monthly but at 200,000 IU.^20^

### Limitations of the study

Firstly, all the patients were one single drug therapy as contrast to international studies where patients were on multiple drugs including insulin. Moreover, the dosage of vitamin D was monthly which can explain the inadequate results of the study as the absorption status of the patients could vary depending upon the complications of diabetes especially autonomic neuropathy which was not evaluated in this study. But its negative results at least give an insight into the fact that prescribing vitamin D should be discouraged in diabetic patients for controlling blood sugar level until compelling study results are available world-wide.

There is no doubt that further studies have to be conducted with different methodology and inclusion and exclusion criteria which could give more insight into the effectiveness of vitamin D in glycemic control of diabetic patients before final verdict can be made.

## CONCLUSION

Vitamin D supplementation can improve the level of Vitamin D in blood but did not have clinically significant impact on HbA1c reduction in Type II diabetic patients.

### Authors’ Contribution

**FAR** conceived, designed and did statistical analysis & editing of manuscript and final approval of manuscript.

**SM, DMK AND SH** did data collection and manuscript writing.
